# DRP1/DMNL-1-mediated mitochondrial fission augments *Rickettsia parkeri* replication in macrophages

**DOI:** 10.1128/iai.00086-26

**Published:** 2026-03-09

**Authors:** Elliott Collins, Natasha Kelly, Heather Green, William Beavers, Basel Abuaita, Juan J. Martinez

**Affiliations:** 1 Vector-Borne Diseases Laboratory, Department of Pathobiological Sciences, Louisiana State University School of Veterinary Medicine 70164, Baton Rouge, Louisiana, USA; 2 Department of Pathobiological Sciences, Louisiana State University School of Veterinary Medicine 70164, Baton Rouge, Louisiana, USA; University of Pennsylvania Perelman School of Medicine, Philadelphia, Pennsylvania, USA

**Keywords:** mitochondria, DRP1, *Rickettsia*

## Abstract

Pathogenic Spotted Fever Group (SFG) *Rickettsia* species, including *Rickettsia parkeri,* replicate in endothelial cells and macrophages *in vitro* and during infections in murine models of disease. We demonstrated that infection of human macrophage-like cells with a related SFG *Rickettsia*, *R. conorii*, resulted in a significant increase in mitochondria-associated proteins. However, the role of mitochondrial functions in *Rickettsia* pathogenesis is unknown. Here, we found that *R. parkeri* exploits mitochondrial dynamics to promote intracellular replication in mouse and human macrophages by activating the mitochondrial fission regulator, the dynamin-related protein 1 (DRP1). *R. parkeri* proliferated in macrophages, which coincided with a significant increase in mitochondria fission, mitochondria content, and host cell ATP production, primarily due to mitochondrial respiration compared to uninfected cells. In addition, *R. parkeri* infection also led to a temporal increase in DRP1 serine phosphorylation that was dependent on rickettsial *de novo* protein synthesis. Importantly, *R. parkeri* growth was significantly impacted in DRP1-deficient macrophages. These results suggest that the modulation of mitochondrial fission, content, and function is important for replication and survival of pathogenic SFG *Rickettsia* species in macrophages. Our data highlight that hijacking mitochondrial dynamics and function is essential for intracellular replication of *Rickettsia* species and may be a shared mechanism utilized by related obligate intracellular pathogens for growth.

## INTRODUCTION

*Rickettsia parkeri* is a Spotted Fever Group (SFG) obligate intracellular Gram-negative human pathogen that is transmitted to humans through tick salivary contents during a blood meal. *R. parkeri* rickettsiosis is found primarily in the southeastern United States and is characterized as a less severe, but important form of rickettsiosis in humans compared to Rocky Mountain spotted fever (RMSF) caused by *Rickettsia rickettsii* (1). In addition, a related SFG *Rickettsi*a species, *R. conorii*, is the agent of Mediterranean spotted fever (MSF), which is endemic to Southern Europe, North Africa, and India (2). Early indications of SFG *Rickettsia* infections in humans are unremarkable and include headache, fever, and malaise. Soon after the tick bite, localized replication of rickettsiae at the inoculation site and ensuing tissue damage may give rise to a necrotic lesion, or eschar. Once established in the host, SFG *Rickettsia* were thought to primarily infect the endothelial lining of the vasculature and cause injury to the vascular endothelium and infiltration of perivascular mononuclear cells leading to vasodilation, an increase in fluid leakage into the interstitial space, and a characteristic dermal rash (3, 4). However, recent data from our group and others have clearly demonstrated that other cell types including monocytes, macrophages, neutrophils, lymphocytes, and hepatocytes are heavily colonized during fatal infections in mammals (5–13). Dissemination of the bacteria in the mammalian host is associated with pulmonary edema, interstitial pneumonia, acute renal failure, liver failure, neurological manifestations, and other multi-organ disorders (14). Broad-spectrum antibiotics such as doxycycline can be used to clinically manage these infections; however, the window of efficacy for these therapies is narrow necessitating the need for the development of more efficacious anti-rickettsial therapies. Therefore, new therapeutic innovations are urgently needed, including host-based approaches to control rickettsial infections.

*R. parkeri* infects recognized target cells such as endothelial cells and can infect circulating monocytes in the peripheral blood and in resident macrophages during infections in murine models of disease (6, 7, 15). PMA-differentiated THP-1 cells have been extensively utilized as model human macrophage cell line for previous studies involving intracellular pathogens such as *Legionella pneumophila* (16), *Coxiella burnetii* (17), *Mycobacterium tuberculosis* (18), and *Chlamydia trachomatis* (19). Using PMA-differentiated THP-1 cells as a model macrophage-like cell, we previously demonstrated that several mammalian pathogenic members of the SFG *Rickettsia* including *R. conorii* (20), *R. rickettsii*, *R. parkeri*, *R. akari,* and *R. africae* are capable of surviving and proliferating within these cells (21). Species that are not associated with disease in mammals, including *R. bellii* and *R. montanensis,* do not replicate in phagocytic cells and are rapidly killed in intracellular compartments containing lysosomal markers (20, 21). These studies further supported the notion that pathogenic *Rickettsia* are not restricted to infecting the vascular endothelium and that other *Rickettsia*-host cell interactions are likely important in pathogenesis.

To initially elucidate host cell pathways that are stimulated by *Rickettsia* species during infection of macrophages, we performed global transcriptome and proteome analyses of *R. conorii*-infected THP-1 macrophages over different time points of infection. These studies revealed that pathways predicted to be involved in establishing a hospitable intracellular environment for growth and survival are modulated to benefit rickettsial growth and survival. Overall, *R. conorii* infection drives the programming of macrophages toward an environment that permits the establishment of a replicative intracellular niche that is characterized, in part, by an accumulation of several enzymes of the tricarboxylic acid (TCA) cycle, oxidative phosphorylation (OXPHOS) fatty acid β-oxidation as well as several inner and outer membrane mitochondrial transporters. Increases in these mitochondrial proteins suggested that pathogenic SFG *Rickettsia* species may induce a stimulation of *de novo* mitochondrial biogenesis (mitogenesis), an increase in the oxidative capacity of mitochondria, and an increase in the β-oxidation of synthesized host cell fatty acids (22, 23). To further support this hypothesis, we previously demonstrated that pharmacological inhibition of host cell *de novo* fatty acid synthesis and reduced expression of mammalian fatty acid synthase (FASN) greatly inhibited rickettsial growth within macrophages. In addition, pharmacological inhibition studies targeting key mammalian cell enzymes and pathways involved in lipid metabolism including triglyceride lipases (inhibited by Orlistat) and fatty acid β-oxidation (inhibited by Etomoxir) revealed that rickettsial growth was significantly diminished in these cells. Taken together, these studies suggested that the modulation of mitochondrial function to potentially provide ATP through OXPHOS and other important mitochondria-derived metabolites are critical events involved in the growth and survival of pathogenic *Rickettsia* species in mammalian phagocytic cells (22–25).

While the important role(s) of modulating mitochondria function in macrophages by several intracellular pathogens including *C. trachomatis*, *S. typhimurium*, *B. abortus*, *L. pneumophila,* and *L. monocytogenes* has been well established (26), very little is known regarding the impact of these processes to the growth and survival of *Rickettsia* species and other obligate intracellular bacteria in macrophages. Murine immortalized bone marrow-derived macrophages (iBMDMs) exhibit characteristics of freshly isolated bone marrow-derived macrophages (BMDMs) *in vitro*, can be propagated indefinitely using the appropriate growth media, and have been successfully utilized to study pathogen-macrophage interactions for several intracellular bacterial pathogens (27). Using iBMDMs as a macrophage model and primary human monocyte-derived macrophages, we showed that *R. parkeri* can efficiently proliferate within these cells. *R. parkeri* infection of iBMDMs and primary human macrophages results in a significant disruption of mitochondrial networks, resembling mitochondrial fission. In addition, *R. parkeri* infection of macrophages results in significant increases in mitochondria content and stimulation of ATP production primarily through mitochondrial respiration. Furthermore, *R. parkeri* infection of iBMDMs leads to the hyper serine phosphorylation of a key regulator of mitochondrial dynamics (DRP1/DNM1L) that is dependent on rickettsial *de novo* protein synthesis suggesting that DRP1-mediated activation of mitochondrial fission may be an important event in the infection process. Indeed, inhibition of *drp1* expression in iBMDMs diminishes the ability of *R. parkeri* to efficiently proliferate within these cells. Together, these results strongly indicate that initiation of DRP1-mediated mitochondrial dynamics plays an important role in the proliferation of *R. parkeri* during infection of mammalian macrophages.

## MATERIALS AND METHODS

### Cell lines, *Rickettsia* growth and purification

African green monkey kidney epithelial cells (Vero) and L929 fibroblast cells were grown at 37°C/5%CO_2_ in Dulbecco’s modified Eagle’s medium (DMEM; Corning) supplemented with 10% heat-inactivated fetal bovine serum (hiFBS, Biowest), 1× non-essential amino acids (Corning), and 0.5 mM sodium pyruvate (Corning). Murine immortalized bone marrow- derived macrophages (iBMDM) were grown in macrophage media (DMEM supplemented with 20% hiFBS and 30% filtered L929 supernatant) as previously described (27). Lentiviral knockdown of *drp1* expression in iBMDMs and isolation of cell clones has been previously described (27). *Rickettsia parkeri* isolate Portsmouth was obtained from Dr. Christopher Paddock (US Center for Disease Control) (28), propagated in Vero cells in a humidified 5% CO_2_ incubator at 34°C and purified as previously described (5, 29, 30). *R. parkeri* was used between passage 2 and passage 4 after being received in our laboratories at the LSU School of Veterinary Medicine.

### Differentiation of human primary macrophages

Human blood samples were obtained from healthy volunteers from the clinical laboratory of Pennington Biomedical Research Center (LSU System, Baton Rouge, LA). The mononuclear cells (PBMCs) fraction was isolated by centrifuging the blood onto a Ficoll-Paque plus density gradient medium (Millipore Sigma, Cat# 17-1440-02). PBMCs were incubated at 37°C in 5% CO_2_ and non-adherent cells were removed by extensive washing with PBS. Adherent cells were used to differentiate monocytes into macrophages by culturing the cells in purified human recombinant M-CSF (50 ng/mL, Biolegend, Cat# 574816) in DMEM supplemented with 20% heat-inactivated fetal bovine serum (FBS) for 6 days. Differentiated macrophages were used at 80% confluency, which was estimated to be 10^5^ cells/well in 24 well plates.

### Antibodies and pharmacological inhibitors

Anti-Rc_PFA_ is a rabbit polyclonal antibody that recognizes multiple species of SFG rickettsiae, including *R. parkeri*, and was previously generated and described (5, 31). Anti-Drp1 rabbit monoclonal antibody (cloneD6C7) was purchased from Cell Signaling Technologies. Anti-Drp1^Ser616^ rabbit polyclonal antibody and anti-Complex I mouse monoclonal antibody (clone 18G12BC2) were purchased from Invitrogen. Alexa Fluor 488-conjugated goat anti-rabbit IgG, Alexa Fluor 546-conjugated goat anti-rabbit IgG, Alexa Fluor 546-conjugated goat anti-mouse IgG, Alexa Fluor 647-conjugated goat anti-mouse IgG, Alexa Fluor 546-phalloidin, Alexa Fluor 488 phalloidin, and DAPI (4′,6′-diamidino-2-phenylindole) were purchased from Thermo Scientific. Alendronate, a nitrogen-containing bisphosphonate (NBP), was purchased from Matrix Scientific (Columbia, SC).

### Analysis of *R. parkeri* growth dynamics

#### Quantitative PCR

Analysis of *R. parkeri* growth in macrophages was performed as previously described with slight modifications (20). Briefly, wild-type iBMDMs, *drp1*-silenced iBMDMs, or primary human macrophages were seeded into 24-well plates in triplicate at a density of 5 × 10^5^ per well. On the day of the experiment, cells were washed once in 1× Dulbecco’s modified phosphate-buffered saline (DPBS) and infected with *R. parkeri* at a multiplicity of infection (MOI) of 5 in prewarmed macrophage media. Plates were centrifuged at 300 × *g* for 5 min at room temperature to induce contact between rickettsiae and host cells and then incubated at 34°C in the presence of 5% CO_2_ for the indicated time points. Cells were harvested by scraping into 1.0 mL of PBS and centrifuged to pellet cells at 900 × *g* for 5 min at room temperature. Total genomic DNA extractions were performed using the PureLink Genomic DNA Mini Kit (Thermo Fisher Scientific) following the manufacturer’s instructions. The growth of *R. parkeri* species determined via a quantitative PCR (qPCR) assay using a LightCycler 480 II (Roche) utilizing the PerfeCTa FastMixII system (QuantaBio) and the following parameters: 10 min at 95°C; 50 cycles of 95°C for 10 s, 58°C for 1 min, and 72°C for 1 s. This was followed by a cool-down cycle lasting 30 s at 40°C. Growth was assessed following the amplification of the rickettsial *sca1* gene and the mammalian *actin* gene by calculating the ratio of *sca1* to *actin* in each well as previously described (20). All experiments were performed using triplicate samples per condition and at least three independent biological experiments.

#### Immunofluorescence microscopy

Immunofluorescence microscopy analysis was also used to verify rickettsial growth within mammalian cells as previously described (20). Briefly, control iBMDMs, *drp1* knockdown iBMDMs, or primary human macrophage cells were seeded at a density of 5 × 10^5^ cells on sterilized glass coverslips in 24-well plates. Cells were infected with *R. parkeri* as described above, washed with 1× PBS, and subsequently fixed with 4% PFA in PBS for 20 min. In some experiments, iBMDMs were preincubated with alendronate (5 µM) for 1 h prior to infection, and the drug was maintained on the cells during the experiment as described (32). *R. parkeri* cells within macrophages were visualized by incubation in antibody incubation buffer (PBS/2% bovine serum albumin [BSA]) containing anti-Rc_PFA_ (1 µg/mL), followed by incubation with either Alexa Fluor 488-conjugated goat anti-rabbit IgG (1:1,000) or Alexa Fluor 546-conjugated goat anti-rabbit IgG (1:1,000). Nuclei and the actin cytoskeleton were visualized by further incubation with DAPI (1:1,000) and Alexa Fluor 488-conjugated phalloidin (1:250) or Alexa Fluor 546 phalloidin (1:250), respectively. All coverslips were then washed in 1× PBS and then mounted onto glass slides using Mowiol mounting medium. Cells were then viewed and imaged using an Olympus Fluoview FV10i confocal microscope with a 40× oil immersion objective. Images were further processed using the Olympus cellSens Dimension software package and the NIH Image J software package and are presented as separate and merged channel images from the “maximum projection” function in the cellSens Dimension software suite.

#### Flow cytometry

Primary human macrophages, wild-type iBMDMs, and *drp1* knockdown iBMDMs were seeded on 6-well plates at a density of 1 × 10^6^ cells/well and incubated for 24 h at 37°C/5% CO_2._ Triplicate wells were left un-infected or infected with *R. parkeri* at a MOI of 5 for 4, 24, and 48 h. In some experiments, iBMDMs were preincubated with alendronate (5 µM) or vehicle control (ddH_2_O) as described above. After each time point, cells were washed in 1× PBS, and each well was scraped into 1.0 mL of 1× PBS. Cells were pelleted by centrifugation at 900 × *g* at room temperature and then incubated in 250 µL of CytoFix/CytoPerm (BD Biosciences) at 4°C for 20 min to fix cells. After fixation, cells were washed twice in 1× Perm/Wash buffer (BD Biosciences) and stored in buffer at 4°C until processing for flow cytometry analysis. To detect *R. parkeri* in infected cells, control uninfected and *R. parkeri*-infected cells were incubated with 1 µg/mL of Anti-Rc_PFA_ antibody for 1 h at room temperature in 1× Perm/Wash buffer. Cells were washed 3× in 1× Perm/Wash buffer and then incubated for an additional hour at room temperature in 1× Wash/Perm buffer containing Alexa Fluor 488-conjugated goat anti-rabbit IgG (1:1,000). Cells were washed in 1× Perm/Wash buffer and analyzed on a BD LSR Fortessa X-20 cytometer equipped with BD FACS DIVA analysis software. At least 10,000 cells were analyzed per sample, and the experiment was performed at least two independent times.

### Confocal microscopy and quantification of mitochondria fragmentation

Primary human macrophages and iBMDMs were seeded at 5 × 10^5^ cells per well in 24-well plates on sterile glass cover slips, allowed to adhere for 24 h at 37°C/5% CO_2_, and then infected with *R. parkeri* at a MOI of 5. Uninfected control cells and *R. parkeri*-infected cells were centrifuged at 300 × *g* for 5 min at room temperature to induce *Rickettsia*-host cell contact and incubated for 24 h at 34°C/5% CO_2_. Mammalian cells were washed with 1× PBS and then fixed in 4% paraformaldehyde (PFA) for 20 min prior to staining. Cells were permeabilized with PBS/0.05% Triton X-100 for 5 min at room temperature and incubated with the primary antibodies rabbit anti-Rc_PFA_ (1 µg/mL) and mouse anti-Complex I (1 µg/mL) followed by the secondary antibodies Alexa Fluor 488-conjugated goat anti-rabbit IgG (1:1,000) and Alexa Fluor 546-conjugated goat anti-mouse IgG (1:1,000) and DAPI. The coverslips were mounted onto slides using Mowiol mounting medium, and images were obtained with the use of an Olympus Fluoview FV10i confocal microscope with a 40× or 100× oil immersion objective. Images were further processed using the Olympus cellSens Dimension software package and the NIH Image J software package.

Mitochondrial fragmentation was quantified using ImageJ software based on similar criteria as previously described (27). Briefly, Complex I immunofluorescence images were converted to binary images, and then objects were outlined. Individual cells were manually selected based on DAPI and Complex I immunofluorescence stains. Mitochondrial objects were categorized as fragmented based on object size between 0.1 and 1 μm^2^. The numbers of fragmented mitochondria from each condition were enumerated per cell from at least 100 cells from three independent experiments.

### DRP1 immunoprecipitation and Western immunoblotting

For immunoprecipitation experiments, *R. parkeri* infections were carried out as described above at an MOI of 5 for the indicated time points with minor modifications. *R. parkeri* cells were pre-incubated with vehicle control or 20 µg/mL chloramphenicol in macrophage media for 1 h at 34°C/5%CO_2_ and then washed in PBS prior to infection of cells as described (25). Infected cells were washed in ice-cold 1× PBS, and 1% NP-40 detergent soluble lysates were generated from uninfected and infected cells as previously described (33). Detergent- soluble proteins were quantified by BCA assay, normalized for protein content, and immunoprecipitated using 2 µg/mL rabbit anti-DRP1 (clone D6C7) and 25 µL of 50% Protein A/G agarose. Immunoprecipitation reactions were washed in 1% NP-40 buffer and boiled in 25 µL of 2× SDS-PAGE sample buffer. Proteins were resolved on 4%–20% mini protein TGX gradient gels (BioRad), transferred to nitrocellulose membranes, and then blocked with 1× Tris-buffered saline (TBST) 2% BSA before incubation with anti-DRP1^ser616^ (2 µg/mL) and anti-rabbit IgG-HRP conjugate (1:25,000). Proteins were revealed by incubation of membranes with SuperSignal West Pico chemiluminescence horseradish peroxidase substrate (Thermo Scientific) and exposure to film. Membranes were stripped with Restore stripping buffer, blocked again in 1× TBST/2%BSA, and reprobed with anti-DRP1 (clone D6C7, 2 µg/mL) and anti-rabbit IgG-HRP conjugate (1:25,000) to ensure equal protein content in each immunoprecipitation reaction. As a further control, generated detergent-soluble lysates were resolved on 4%–20% mini protein TGX gradient gels, transferred to nitrocellulose, and immunoblotted with anti-DRP1 (clone D6C7, 2 µg/mL) and anti-rabbit IgG-HRP conjugate as described above. Depicted western blots are representative of at least two independent biological experiments.

### Seahorse analysis

The rate of ATP production from glycolysis (glycoATP) and mitochondrial respiration (mitoATP) was measured by Seahorse XF Real-Time ATP Rate Assay (Agilent, Cat# 103677-100) as previously described (27). NT-control and Drp1 KD macrophages were seeded onto Seahorse XF96 cell culture microplates at a density of 4 × 10^4^ per well and cultured at 37℃ for 24 h. Media was replaced, and macrophages were left untreated (Mock) or infected with *R. parkeri* for 6, 24, and 48 h. As a control, macrophages were incubated with lipopolysaccharide (LPS; 500 ng/mL) for 6 h to stimulate glycolytic pathways. During the last hour of the experiment, macrophages were washed with the assay culture media and placed in a non-CO_2_ incubator at 37℃ for 45 min. Media were further exchanged with fresh and warmed assay media. Oxygen consumption and extracellular acidification were monitored using the Seahorse XFe96 Analyzer. Data were analyzed by using the XF Real-Time ATP Rate Assay Report Generator to quantify the rate of ATP.

### Mitochondrial DNA quantification

Macrophages were left uninfected or infected with *R. parkeri* for the indicated time points, were harvested by scraping, and resuspended into 200 μL of phosphate saline buffer. Total DNA was isolated using the DNeasy Blood and Tissue Kit (Qiagen). Nuclear and mitochondrial DNA in the samples were quantified by real-time PCR using the following primers: 18S forward (5′-TAGAGGGACAAGTGGCGTTC-3′); 18S reverse (5′-CGCTGAGCCAGTCAGTGT-3′); Cytochrome c oxidase 1 (MT-CO1) forward (5′-GCCCCAGATATAGCATTCCC-3′); MT-CO1 reverse (5′-GTTCATCCTGTTCCTGCTCC-3′). The abundance of mitochondrial DNA (MT-CO1) levels was normalized relative to untreated (Mock) macrophages and presented as a ratio. The Ct value of MT-CO1 was divided by the Ct value of 18S for each sample. Then, the relative abundance of mitochondrial DNA (MT-CO1) levels was calculated relative to the average ratio of MT-CO1/Nuclear of uninfected samples.

## RESULTS

### *R. parkeri* proliferates within murine and primary human macrophages

**Fig 1:**
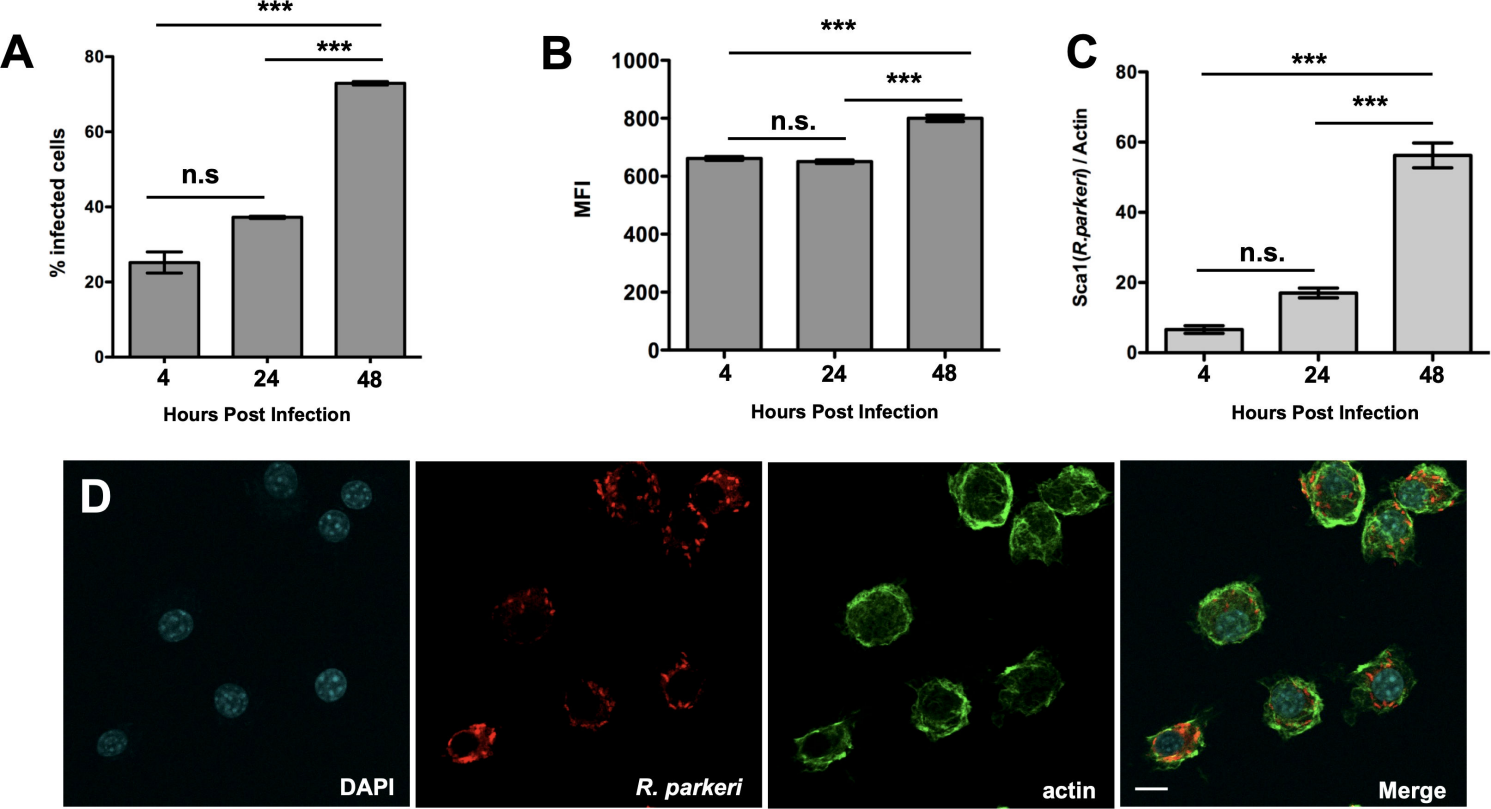
*R. parkeri* strain Portsmouth proliferates in immortalized murine bone marrow-derived macrophages (iBMDMs). Flow cytometry analyses demonstrate an increase in the percentage of cells infected and a corresponding increase in the mean fluorescence intensity (MFI) of *R. parkeri* infected cells over time (**A and B**). Q-PCR analysis confirms an increase in *R. parkeri* growth as a ratio of rickettsial genomic content (*sca1*) over mammalian cell content (*actin*) (**C**). *R. parkeri* infected iBMDMs at 48 h post-infection reveal intact bacteria within iBMDMs. Actin (green), *Rickettsia* (red), nuclei (blue) (**D**). Statistically significant changes were determined using a one-way analysis of variance (ANOVA) with Bonferroni’s comparison *post hoc* test. n.s. = not significant, ^***^P < 0.0001. Scale bar = 5 µm in D.

**Fig 2:**
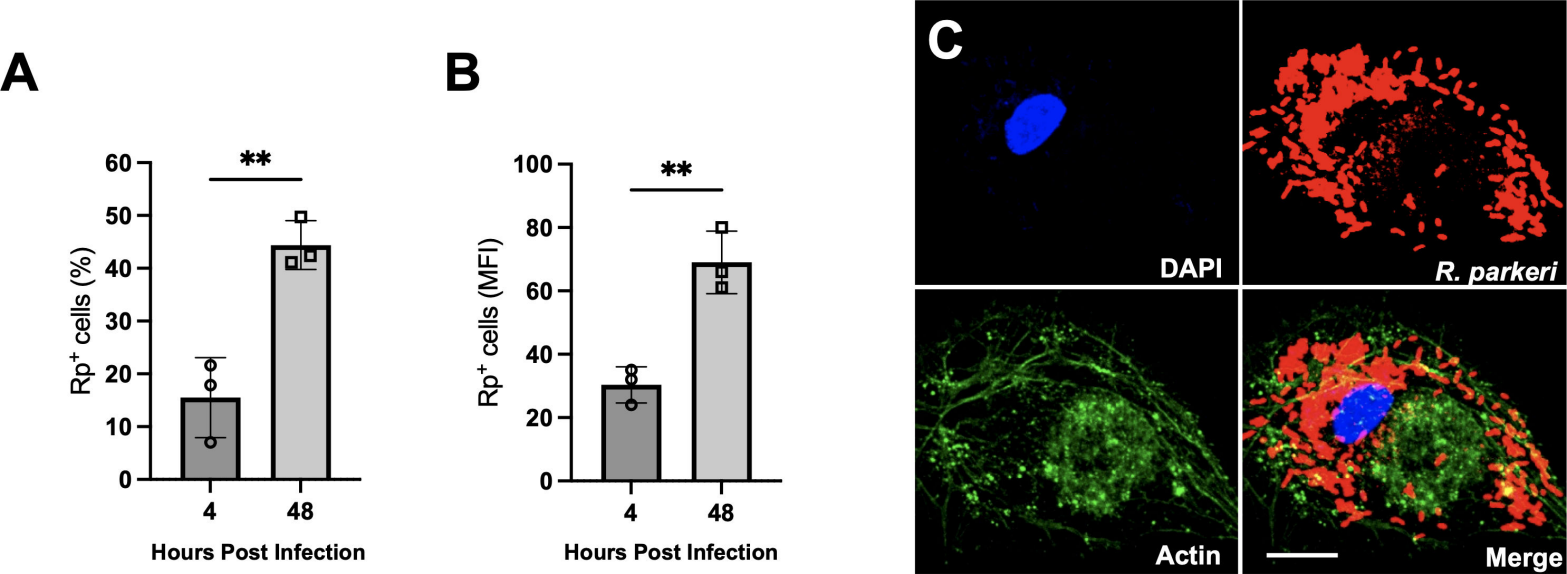
*R. parkeri* proliferates within primary human monocyte-derived macrophages. (**A and B**) Flow cytometry analysis of *R. parkeri* growth in human primary macrophage demonstrates an increase in the percent of infected cells in the population (**A**) and *Rickettsia* specific fluorescence (mean fluorescence intensity, MFI) (**B**). Fluorescence microscopy analysis of *R. parkeri* infected human primary macrophages at 48 h post-infection (**C**). DAPI (blue), *R. parkeri* (red), actin (green). Scale bar = 5 µm. Statistically significant changes were determined via a Mann-Whitney test. ^**^P < 0.001.

We have previously demonstrated that SFG *Rickettsia* species, including *R. conorii*, *R. prowazekii*, and *R. parkeri* strain “Portsmouth” can efficiently invade into and proliferate within THP1-differentiated cells as a model for mammalian macrophages (21). However, the differentiation of THP-1 cells into macrophage-like cells requires stimulation with Phorbol Myristate Acetate (PMA), which may activate the cells and alter *Rickettsia* replication. Thus, we investigated whether murine immortalized bone marrow-derived macrophages (iBMDMs) can be used as an alternative macrophage model to study *Rickettsia* pathogenesis. These iBMDMs are an established model mammalian macrophage and have been previously utilized for the study of microbial pathogenesis and host defenses (27). We first sought to determine whether *R. parkeri* could invade into and replicate within the iBMDMs. The cells were infected with *R. parkeri,* and bacterial burden was measured by flow cytometry-based assay using anti-*R*. *parkeri* antibody. *R. parkeri* infection leads to a significant increase in the percent of infected macrophages and the mean fluorescence intensity over the time course of the experiment, indicating that the bacteria invade and replicate within these cells (Fig. 1A and B). We commonly use the quantitative PCR (qPCR)-based assay that directly measures *R. parkeri* DNA relative to host genomic DNA as indicative of intracellular bacterial loads (34, 35). To establish whether the increase in fluorescence signals using the anti-*R*. *parkeri* antibody correlates with an increase in rickettsial loads, we utilized the qPCR-based growth assay to confirm *R. parkeri* growth within these macrophages (Fig. 1C). Similar to the flow cytometry data, we observed an increase in *R. parkeri* DNA over time, indicating that *R. parkeri* is replicating within the iBMDMs. We next visualized the infected cells by confocal microscopy and observed an increase in bacterial loads, especially at 48 h post-infection (Fig. 1D). Using the flow cytometry and fluorescence microscopy-based assays, we also confirmed that *R. parkeri* efficiently proliferates within primary human macrophages and that growth was not restricted to macrophages of murine origin (Fig. 2). Taken together, these results confirm that the iBMDMs can be utilized as a model for the study of *Rickettsia*-macrophage interactions and that *R. parkeri* can efficiently proliferate within the mouse and human macrophages.

### *R. parkeri* stimulates changes to macrophage mitochondrial architecture

**Fig 3:**
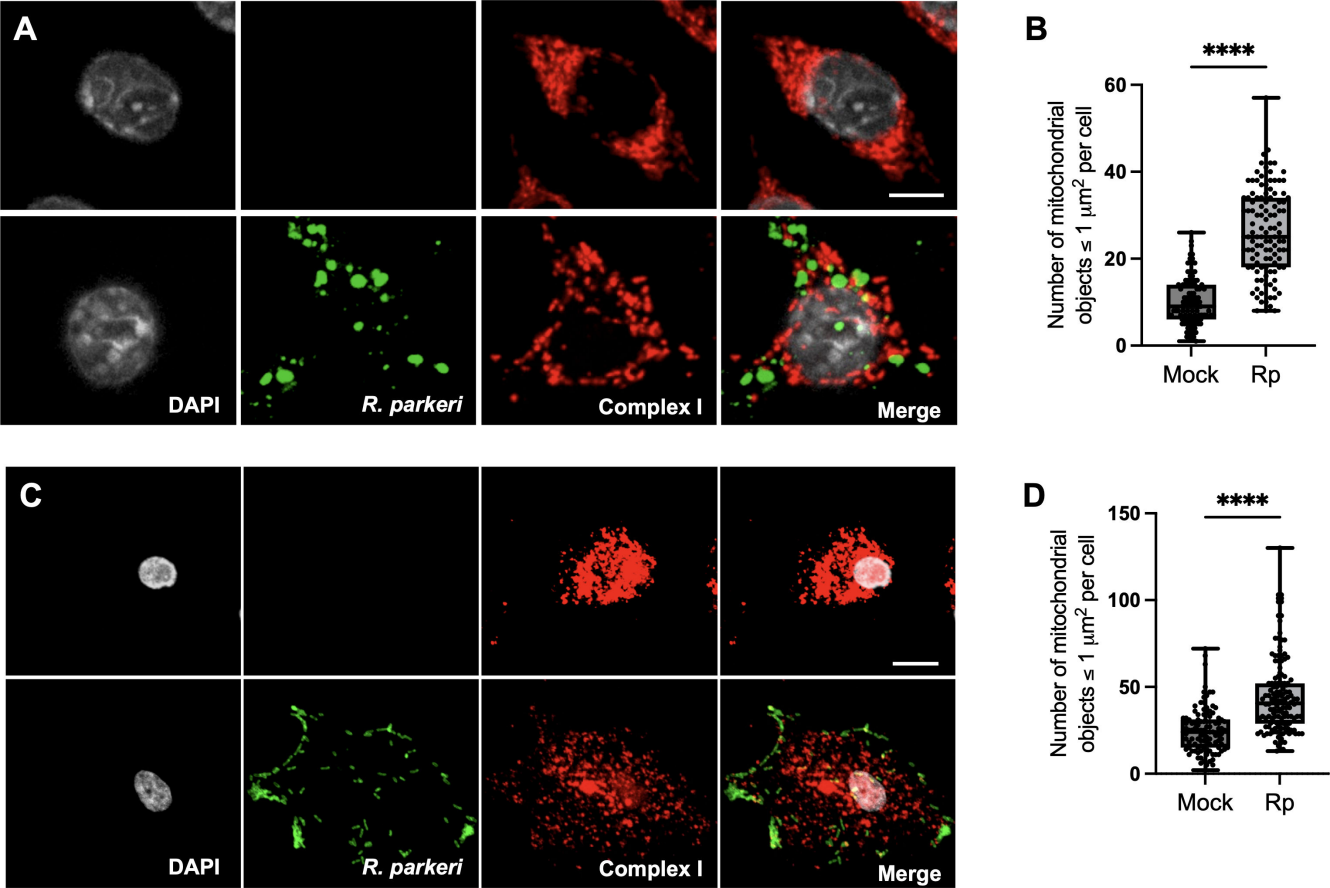
*R. parkeri* induced mitochondrial dynamics in mammalian macrophages. Infection of iBMDMs and primary human macrophages drives significant changes in mitochondria morphology at 48 h post infection. (**A**) Representative confocal microscopy images of iBMDM (**B**) and primary human macrophages (**C**) when left uninfected (upper panels) or infected with *R. parkeri* (lower panels) for 48 h. Cells were stained with anti-RcPFA antibody (green), anti-Complex I antibody (red), and DAPI (gray) to label the DNA. Scale bar in (**A**) = 5 µm while the scale bar in C = 10 µm. Quantification of mitochondrial fragmentation in iBMDMs (**B**) and primary human macrophages (**D**) was performed on Complex I immunofluorescence images by using ImageJ software. The number of mitochondrial objects with a size between 0.1 and 1 μm² was enumerated per cell. A graph is presented as a box and whisker plot, showing the minimum and maximum number of mitochondrial objects per cell from at least 100 cells across *n* ≥ 3 independent experiments. Statistical significance was calculated using the Mann-Whitney test. ^****^P < 0.0001.

Infection of differentiated THP-1 macrophage-like cells with a related SFG *Rickettsia* species, *R. conorii*, led to a significant increase in several enzymes involved in the tricarboxylic acid (TCA) cycle, oxidative phosphorylation (OXPHOS), fatty acid β-oxidation, and glutaminolysis, as well as proteins involved in mitochondrial function (23). However, the beneficial consequences of altering these processes to *R. parkeri* replications remained unclear. Several intracellular bacterial pathogens, including *L. pneumophila* (vacuolar pathogen) and *L. monocytogenes* (cytoplasmic pathogen), manipulate the infected host cell mitochondrial network to promote intracellular replication. The function of mitochondria is influenced by changes in organelle morphology, quantity, and often localization. To determine if pathogenic *Rickettsia* species can also stimulate changes to mitochondrial morphology, we infected murine and primary human macrophages with *R. parkeri* for 48 h and assessed mitochondrial dynamics by immunofluorescence microscopy using anti-complex I antibody. *R. parkeri* infected mouse macrophages exhibited an increase in mitochondria fragmentation (fission) that had been previously observed for *L. pneumophila* in macrophages (36). Similar results were also observed in primary human macrophages infected with *R. parkeri* for 48 h (Fig. 3C and D). These results suggest that the modulation of mitochondria morphology may play an important role during infection of SFG *Rickettsia* species in mammalian macrophages.

### *R. parkeri* drives increases in mitochondria content and mitochondria respiration in infected macrophages

**Fig 4:**
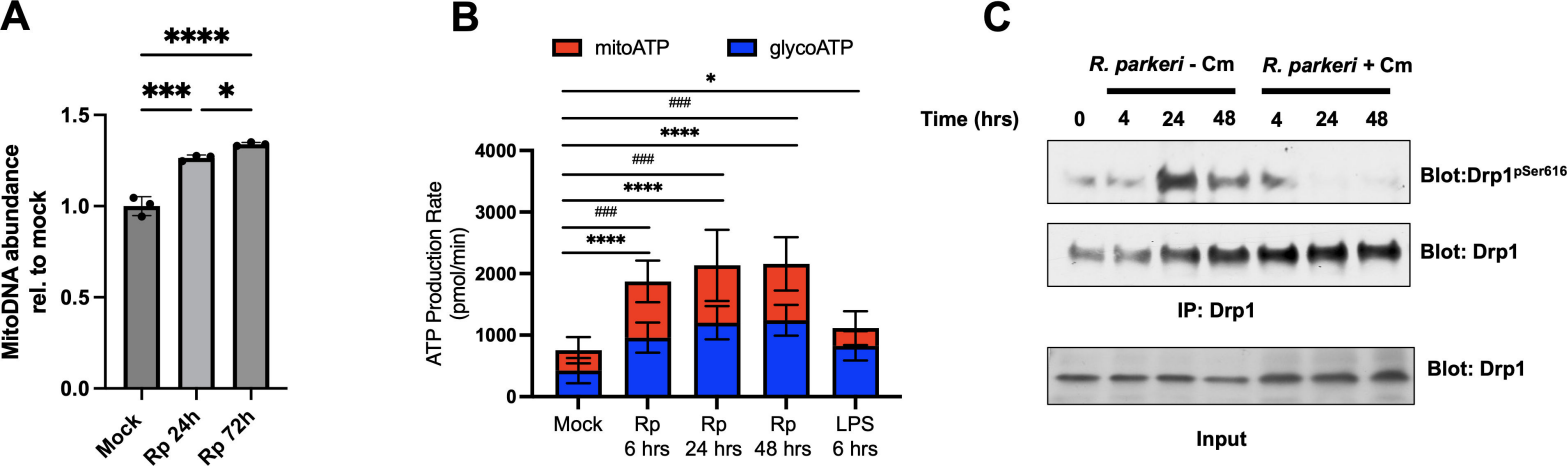
*R. parkeri* infection drives *de novo* mitochondrial biosynthesis and stimulates increases in ATP production via mitochondrial respiration and DRP1 serine phosphorylation. (**A**) qPCR analysis of uninfected (mock) or *R. parkeri* infected cells at 24 h (Rp 24 h) and 72 h (Rp 72 h) reveals a significant increase in mitochondrial DNA. Statistical significance was determined via a one-way ANOVA analysis with Bonferroni’s comparison post-hoc test, ^*^P = 0.0286, ^***^P = 0.0001, and ^****^P < 0.0001. (**B**) Seahorse analysis of ATP production revealed significant changes in both glycolysis (blue) and mitochondrial respiration (red) at indicated times post-infection. LPS drives maximal ATP production via glycolysis and is used as a control. Statistical significance was established using a two-way ANOVA and Holm-Sidaks multiple comparison test. Only significant differences are indicated, ^*^P = 0.0386, ^###^*P* = 0.0002, ^****^P < 0.0001. (**C**) Macrophages were infected for the indicated time points with *R. parkeri* left untreated (*R. parkeri*-Cm) or pre-treated with chloramphenicol (*R. parkeri* +Cm) at an moi = 5. Detergent-soluble protein lysates were generated, and equal amounts of total proteins were immunoprecipitated with an anti-DRP1 antibody (IP:Drp1). Transferred proteins were probed with an anti-DRP1^pSer616^ antibody and re-probed with an anti-DRP1 antibody. To control for protein loading, generated lysates were probed with anti-DRP1 antibody (input).

Infection of THP-1 macrophages with *R. conorii* led to a significant increase in the abundance of mitochondrial transporters including voltage-dependent ion channels 1–3 (VDAC1–3), solute carrier family 25 members 1, 3, 5, and 6 (SLC25A1, 3, 5, 6), translocase of outer mitochondrial membrane 22 and 40 (TOMM22 and TOMM44) along with an increase in proteins from Complex I (NDUFB8), Complex II (SDHB), Complex III (UQCRC2), and Complex IV (CoxII) (23). Increases in these mitochondrial proteins suggested that infection of phagocytic cells with a pathogenic SFG *Rickettsia* species would result in a stimulation of *de novo* mitochondrial biosynthesis (mitogenesis) and a corresponding increase in the oxidative capacity of mitochondria in infected cells. To determine if the previously observed changes in mitochondria-associated protein levels induced by SFG *Rickettsia* infection correspond to an increase in mitochondria content, we infected macrophages with *R. parkeri* and measured the levels of mitochondrial DNA at 24 and 72 h. Mitochondrial DNA in uninfected and infected cells was quantified by RT-qPCR relative to nuclear DNA as previously described (37). As shown in Fig. 4A*, R. parkeri* infection in iBMDMs leads to a significant increase in mitochondrial DNA abundance relative to uninfected cells at 24 and 72 h post infection, suggesting that mitochondrial contents increase during *R. parkeri* infection.

We further hypothesized that infection of mammalian macrophages by pathogenic *Rickettsia* species will result in significant and dynamic changes in ATP production to potentially fulfill metabolic requirements of this class of obligate intracellular pathogens. To test the hypothesis, we monitored ATP levels produced by the mitochondria and glycolysis during infection using the Seahorse Real-Time ATP Rate Assay. The assay calculates mitochondrial ATP (mitoATP) and glycolytic ATP (glycoATP) by measuring oxygen consumption rate and extracellular acidification rate of live cells. Macrophages were infected with *R. parkeri,* and ATP production was quantified at 6, 24, and 48 hpi (Fig. 4B). As a control, macrophages were stimulated with lipopolysaccharide (LPS) to induce immunometabolism shift from mitochondria to glycolysis ATP production. *R. parkeri* induced a modest but statistically significant ATP production via glycolysis when compared to uninfected (mock) cells, indicating *R. parkeri* increases glycolysis. Interestingly, significant mitoATP was also increased as early as 6 hpi and remained elevated at 24 and 48 h. This was different than the LPS-stimulated macrophages where mitoATP levels slightly decreased relative to uninfected cells. These results suggest that *R. parkeri* infection in macrophages leads to a significant increase in mitochondrial metabolism that may play a role in the establishment of a niche suitable for replication.

### *R. parkeri* infection results in DRP1 serine phosphorylation

Activity of DRP1/DNM1L is regulated by phosphorylation of serine residues including Ser616, which can be assayed by western immunoblotting (36). To initially determine if *R. parkeri* can also stimulate DRP1 activation, we infected iBMDMs for the indicated time points, solubilized mammalian proteins, and analyzed the DRP1 phosphorylation state using immunoprecipitation and western immunoblotting with commercially available DRP1-antibodies and DRP1-phosphospecific antibodies. As shown in Fig. 4C, infection of iBMDMs with *R. parkeri* cells leads to a rapid increase in DRP1-Ser616 phosphorylation at 4 h post-infection that is maintained up to 48 h post-infection. Interestingly, *L. pneumophila* utilizes its type IV secretion machinery to secrete the effector proteins (MitF/LegA), inducing DRP1 serine phosphorylation, mitochondrial fragmentation, and ultimately aiding in the formation of a replicative intracellular niche (36). To determine whether *de novo* rickettsial protein synthesis is involved in DRP1 activation, we pre-incubated *R. parkeri* cells with chloramphenicol or vehicle control (25) infected iBMDMs for the indicated time points and processed the isolated soluble protein samples for immunoprecipitation and western immunoblotting analysis. As shown in Fig. 4C, chloramphenicol treatment inhibits *R. parkeri* induced DRP1 serine phosphorylation after 4 h. These results demonstrate that *R. parkeri* can stimulate DRP1 activity during infections of macrophages and that this activation is dependent on *R. parkeri* protein synthesis.

### Silencing DRP1 expression results in decreased *R. parkeri* growth in macrophages

**Fig 5:**
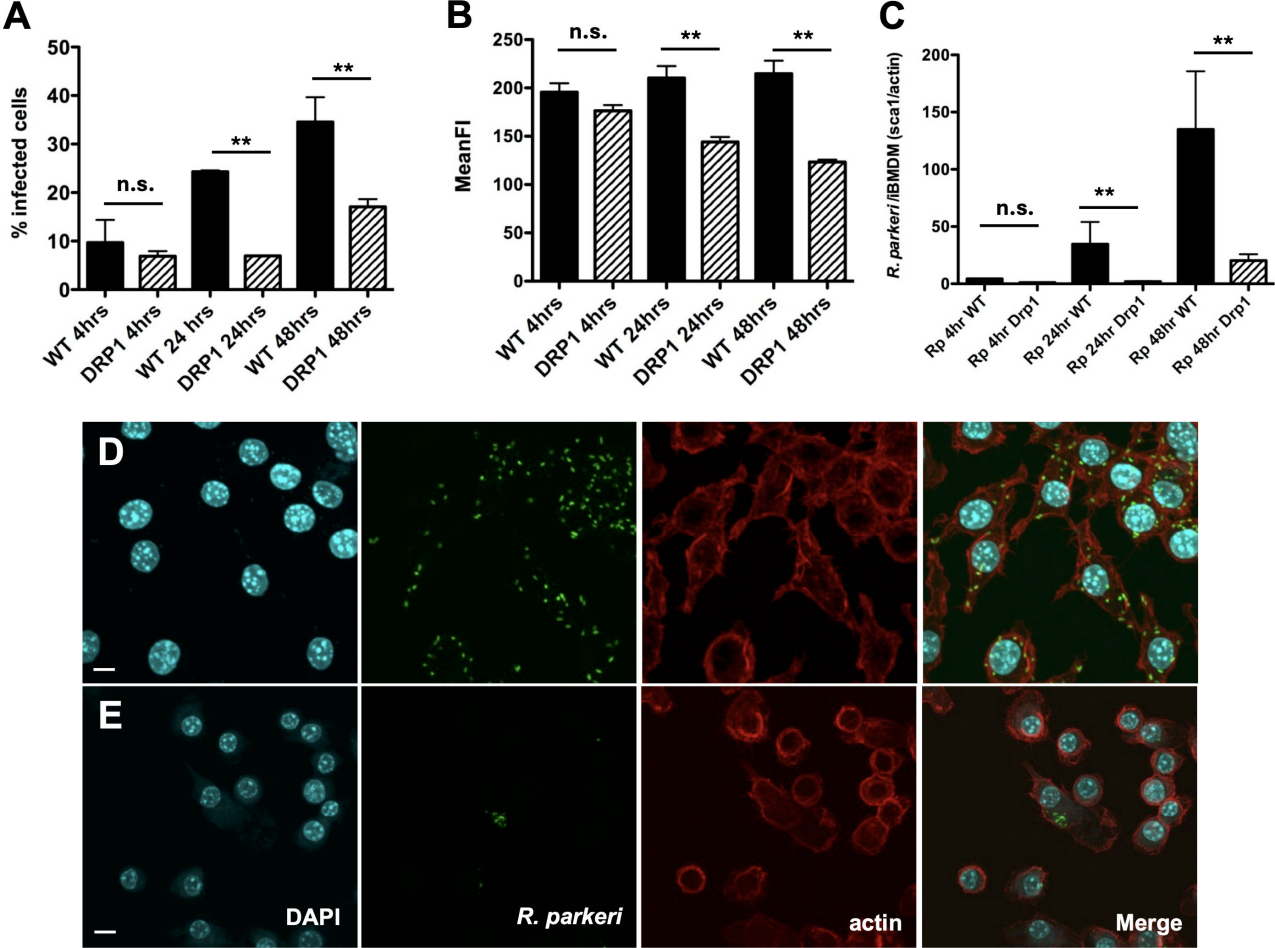
Inhibition of DRP1 expression in iBMDMs diminishes *R. parkeri* proliferation. (**A and B**) Flow cytometry analysis of wild type (WT) and *drp1* knockdown (DRP1) infected cells and the mean fluorescence intensity (MFI) of *R. parkeri* indicated time points. (**C**) Quantitative PCR (qPCR) analysis of WT and DRP1 iBMDMs infected with *R. parkeri* presented as a ratio of *Rickettsia* genomic DNA content (*sca1*) vs mammalian genomic DNA content (*actin*). (**D and E**) Immunofluorescence microscopy analysis of *R. parkeri* WT-infected iBMDMs (**D**) and DRP1-infected iBMDMs (**E**) demonstrates a significant growth inhibition in DRP1 knockdown cells. Scale bar in D and E = 5 µm. Statistical significance was determined using a one-way ANOVA with Bonferroni’s comparison test. n.s = not significant, ^**^P < 0.0001 in A and B, ^**^P = 0.0007 in C.

Our previous study demonstrated that silencing DRP1 reduces mitochondrial fragmentation in LPS-stimulated macrophages, suggesting that DRP1 is essential for altering mitochondrial dynamics during infection (38). In addition, interfering with expression and activity of DRP1 reduces intracellular replication of *L. pneumophila* and *L. monocytogenes* (36) (39), suggesting that mitochondrial fragmentation/fission can favor bacterial replication. Thus, we hypothesized that the observed dynamic changes to mitochondria and DRP1 Ser616 phosphorylation in *R. parkeri*-infected macrophages would also favor the establishment of a replicative intracellular niche. To determine whether DRP1 activity plays a role in *R. parkeri* replication in macrophages, we infected control and DRP1-knockdown iBMDMs with *R. parkeri* and analyzed bacterial loads at 4, 24, and 48 h. As shown in Fig. 5, *R. parkeri* growth was significantly reduced in DRP1 knockdown cells compared to control transfected cells, suggesting that DRP1 is required for *R. parkeri* replication. A recent study implicated key enzymes in the mevalonate pathway as playing key roles in directly influencing mitochondrial dynamics. Nitrogen-containing bisphosphonates (NBPs such as alendronate and zolendronate) inhibit farnesyl diphosphate synthase (FPP) and result in the inhibition of the biosynthesis of cholesterol, isoprenoids, heme, and ubiquinones (40). In addition, pharmacological inhibition of an enzyme, HMG-CoA reductase, upstream of FPP activity reduced *R. conorii* (34) and *R. parkeri* (41) growth with infected mammalian cells. Interestingly, targeting of FPP by NBPs led to a decrease in mitochondrial fission markers in mammalian endothelial cells (32). To determine whether NBPs could potentially inhibit rickettsial growth in macrophages, we pre-incubated iBMDMs with alendronate (5 µM), infected these cells with *R. parkeri,* and determined growth using flow cytometry and immunofluorescence microscopy. Alendronate does not significantly impact the growth of *R. parkeri* in macrophages suggesting that FPP-dependent pathways may not be directly involved in rickettsial growth in phagocytic cells (Fig. S1). Taken together, our results demonstrate that *R. parkeri* induces DRP1 phosphorylation, which is required for efficient *R. parkeri* proliferation independent of NBPs within infected mammalian macrophages.

## DISCUSSION

As obligate intracellular pathogens, several *Rickettsia* species have evolved strategies to infect different mammalian cell types including endothelial cells, monocytes, and macrophages and to sequester nutrients within these infected cells required to establish productive replicative niches. Genomic and bioinformatic analysis of different *Rickettsia* species demonstrated that this class of obligate intracellular bacteria lacks a full arsenal of genes encoding enzymes that are vital to synthesize certain fatty acids and to efficiently produce ATP (42, 43), strongly suggesting that *Rickettsia* species must exploit host cell pathways to obtain these and other host-derived nutrients to fulfill metabolic requirements. Infection of THP-1 macrophages with *R. conorii* led to the predicted upregulation of pathways associated with *de novo* host cell fatty acid biosynthesis and β-oxidation of these fatty acids, presumably for the generation of ATP and changes in mitochondria abundance and function (23). In addition, rickettsial infections of THP-1 macrophages also led to an increase in the abundance of proteins involved in mitochondria function and a decrease in the abundance of several enzymes involved in host cell glycolysis, including phosphoglycerate kinase 1 (PGK1), pyruvate kinase (PKM), and enolase 1 (ENO1), strongly suggesting that rickettsial infections reprogram host cell metabolism to favor oxidative phosphorylation (OXPHOS) over glycolysis (23). In this current study, we demonstrated that infection of mammalian macrophages (iBMDMs) with *R. parkeri* leads to a significant increase in mitochondria content and an increase in mitochondria fission in infected cells during the time course of the experiment. These induced changes coincided with an increased synthesis of host cell ATP primarily through mitochondrial respiration (OXPHOS). In contrast, infection of human monocyte-derived macrophages by the vacuole-bound pathogen, *L. pneumophila*, results in an overall increase in host cell glycolysis and a decrease in OXPHOS (Warburg-like metabolism) along with changes in mitochondrial morphology to establish a more replicative favorable intracellular niche (36). These results strongly suggest that the metabolic requirements between intracellular Gram-negative bacterial pathogens may be distinct and possibly dictated by their respective intracellular localizations and mechanisms utilized to successfully proliferate within target mammalian cells.

Mitochondrial dynamics including mitochondrial fission are controlled in large part by DRP1/DNM1L, a small GTP-binding protein that is activated by serine phosphorylation of a key residue (Ser616) (44). We demonstrated that *R. parkeri* protein synthesis is required to stimulate DRP1 serine phosphorylation starting at 4 h post-infection and maintained until 48 h post-infection, strongly suggesting that DRP1 is activated during the infection. *L. pneumophila* uses its type IV secretion machinery to secrete the effector protein (MitF/LegA) which is involved in DRP1-dependent mitochondrial fragmentation (37). Interestingly, several *R. parkeri* secreted proteins termed “secreted *Rickettsia* factors” (SrfsA-G) have recently been shown to interact with intracellular compartments and organelles, including the host cytoplasm, mitochondria, and endoplasmic reticulum *in vitro*. One of these proteins, SrfB, co-localizes with mitochondrial apoptosis inducing factor (AIF); however, SrfB was not sufficient to mediate significant changes to the mitochondrial networks (45) observed in our study. The putative rickettsial protein(s) that are involved in mediating changes to mitochondrial morphology and in stimulating DRP1 activation in infected macrophages are not known and are currently under investigation.

The enzyme, farnesyl diphosphate synthase (FPP), can play a role in modulating mitochondrial dynamics and the abundance of mitochondria fission proteins in mammalian cells (32). Therefore, targeting FPP could potentially impact *R. parkeri* growth by perturbing mitochondrial function during infection. However, pharmacological inhibition of FPP did not significantly diminish the ability of *R. parkeri* to proliferate within murine macrophages, suggesting that rickettsial growth in these cells is independent of this arm of the mevalonate pathway. The host cell pathways that are stimulated by *R. parkeri* infection and lead directly to DRP1 activation and mitochondrial fission are not yet defined and are currently under investigation. Further elucidation of the host cell pathways which are involved in mitochondrial fission during infection may represent a novel alternative strategy for therapeutic intervention against pathogenic *Rickettsia* species.

## SUPPLEMENTAL MATERIAL

Fig. S1 iai.00086-26-s0001.pdf NBP fails to inhibit *R. parkeri* growth in mammalian macrophages.

Supplemental material iai.00086-26-s0002.docx Fig. S1 caption.

ASM does not own the copyrights to Supplemental Material that may be linked to, or accessed through, an article. The authors have granted ASM a non-exclusive, world-wide license to publish the Supplemental Material files. Please contact the corresponding author directly for reuse.
