# Supplementary figures and images for: DRP1/DMNL-1-mediated mitochondrial fission augments Rickettsia parkeri replication in macrophages
Source: Infect Immun. 2026 Mar 9;94(4):e00086-26. doi: 10.1128/iai.00086-26 (PMC13081717; doi:10.1128/iai.00086-26)

**A**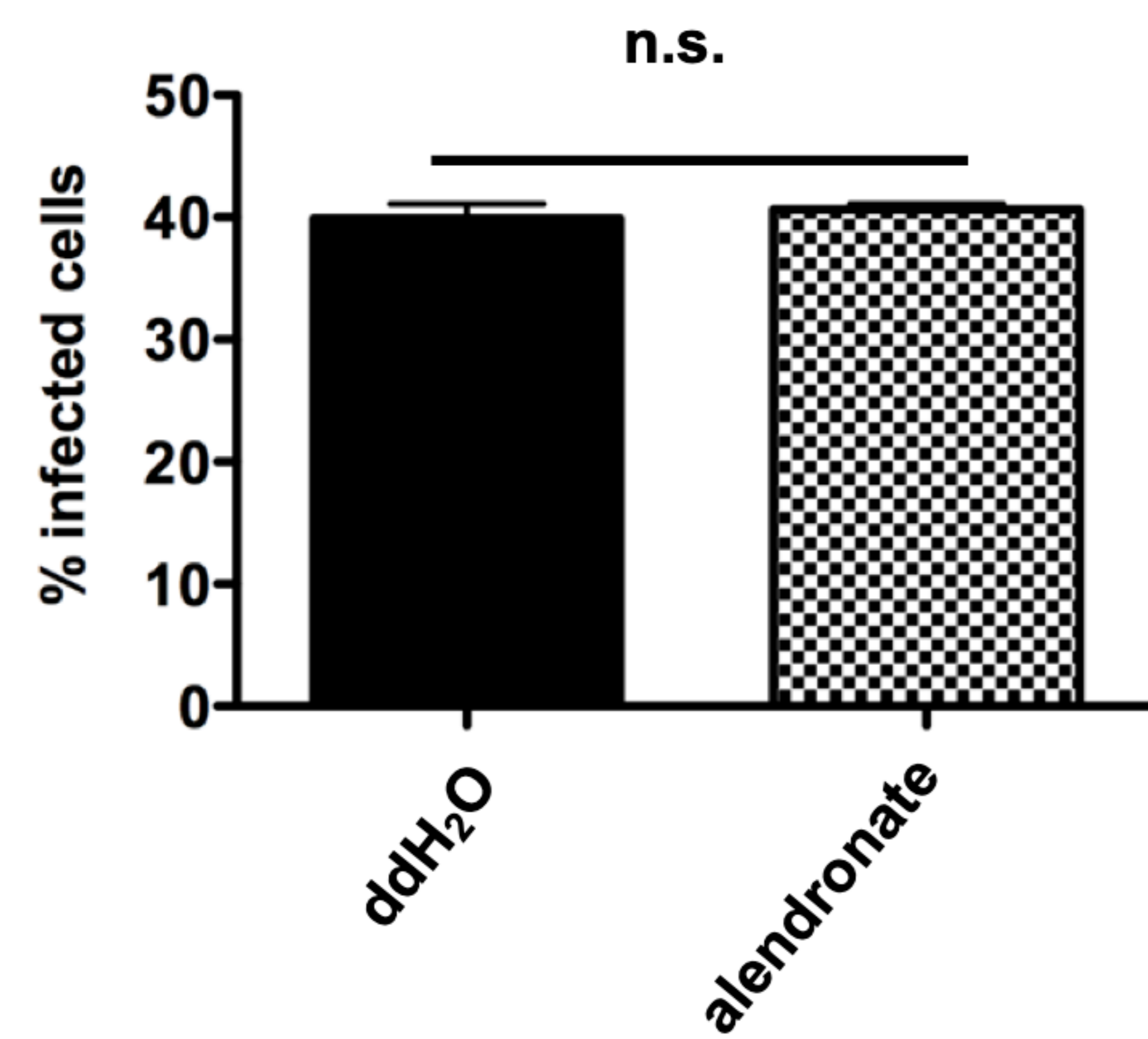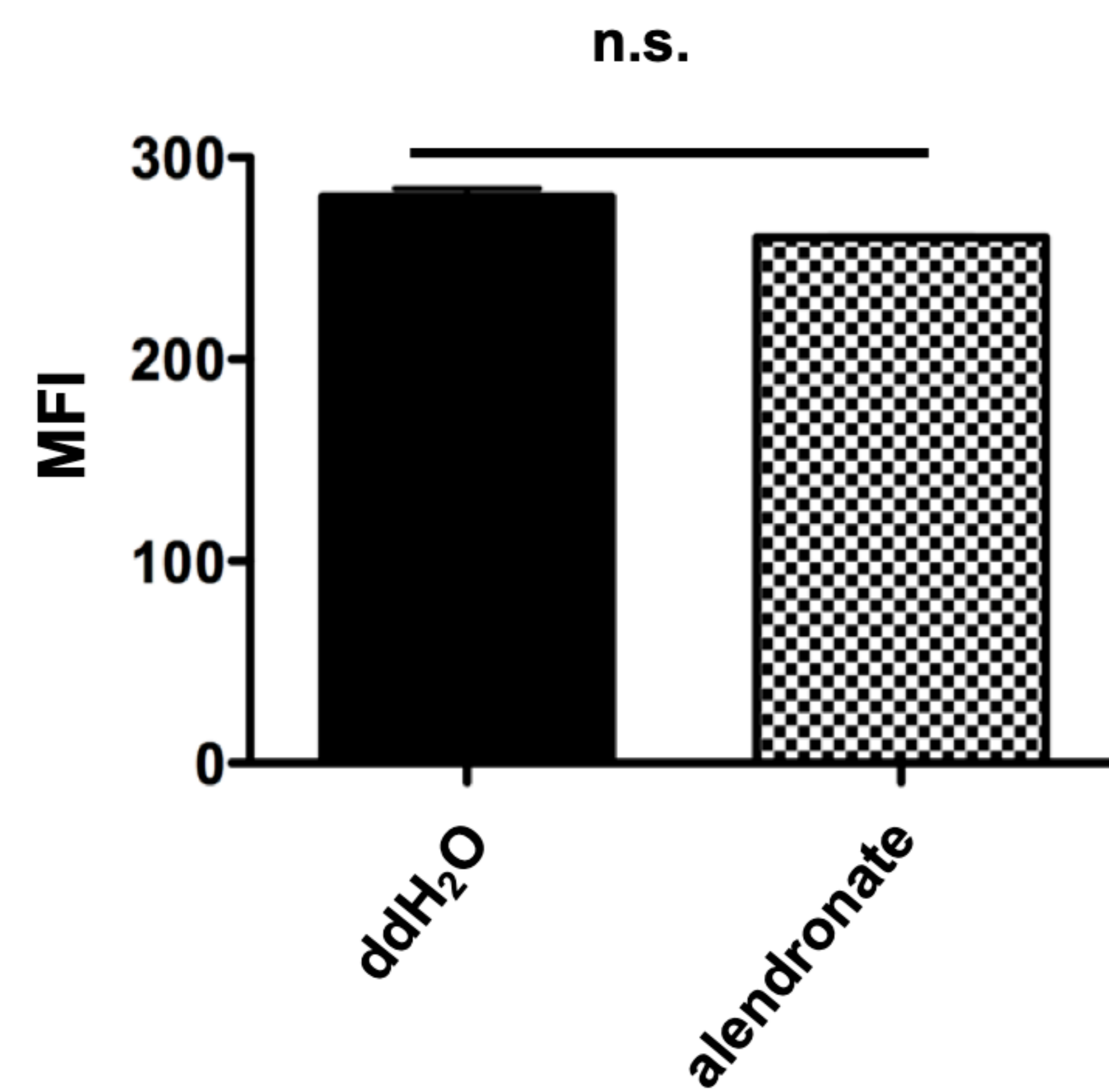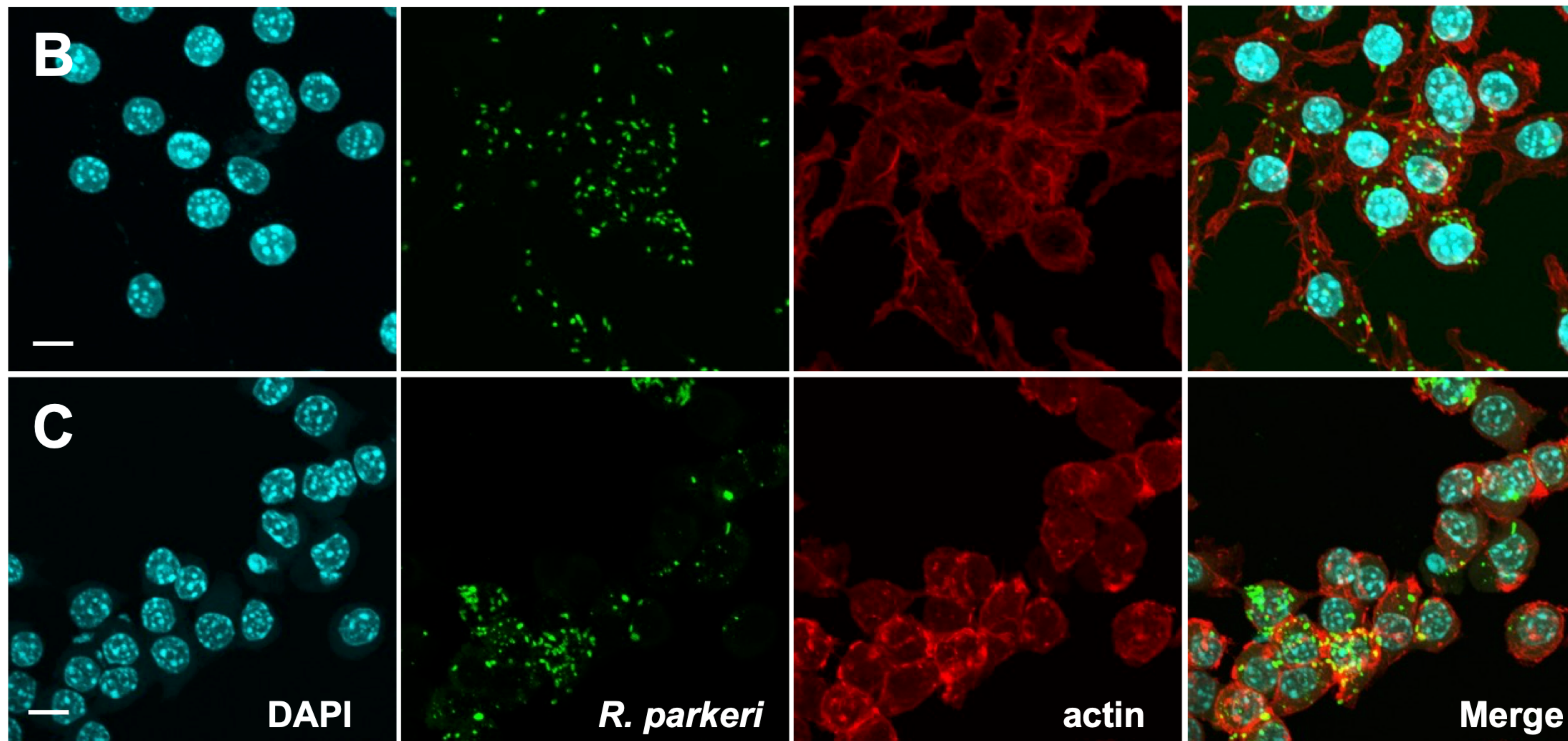

Supplement: Fig. S1 — NBP fails to inhibit R. parkeri growth in mammalian macrophages. [file iai.00086-26-s0001.pdf]
