# Supplementary material for: DRP1/DMNL-1-mediated mitochondrial fission augments Rickettsia parkeri replication in macrophages
Source: Infect Immun. 2026 Mar 9;94(4):e00086-26. doi: 10.1128/iai.00086-26 (PMC13081717; doi:10.1128/iai.00086-26)
Supplement: Supplemental material — Fig. S1 caption. [file iai.00086-26-s0002.docx]

**Supplemental Figure 1. Nitrogen containing bisphosphonates (NBP) fail to inhibit**

716 ***R. parkeri* growth in mammalian macrophages.** (A) Flow cytometry analysis of

717 control-treated (ddH2O) and alendronate-treated (5$\mu$M) iBMDMs infected with *R.*

718 *parkeri* for 48hrs does not reveal significant differences in the percentage of cells

719 infected (% infected cells) nor differences in *Rickettsia* fluorescence signal in infected

720 cells (MFI). Statistical significance was determined using an unpaired t-test, n.s.=not

721 significant. (B and C) Immunofluorescence microscopy analysis reveals similar numbers

722 of bacteria in control (B) and alendronate-treated cells (C). DAPI (blue), *R. parkeri*

723 (green), actin (red). Scale bar =5

$\mu$m.
